# Conventional MRI Criteria to Differentiate Progressive Disease From Treatment-Induced Effects in High-Grade (WHO Grade 3–4) Gliomas

**DOI:** 10.1212/WNL.0000000000200359

**Published:** 2022-07-05

**Authors:** Christina M. Flies, Karlijn H. van Leuken, Marlies ten Voorde, Joost J.C. Verhoeff, Filip Y.F. De Vos, Tatjana Seute, Pierre A. Robe, Theodoor D. Witkamp, Jeroen Hendrikse, Jan Willem Dankbaar, Tom J. Snijders

**Affiliations:** From the Department of Neurology & Neurosurgery, UMC Utrecht Brain Center (C.M.F., K.H.v.L., M.t.V., T.S., P.A.R., T.J.S.), and Departments of Radiation Oncology (J.J.C.V.), Medical Oncology (F.Y.F.D.V.), and Radiology (T.D.W., J.H., J.W.D.), University Medical Center Utrecht; Stichting Beroepsopleiding Huisarts (K.H.v.L.), the Netherlands; and Mission of the Netherlands Reformed Congregations in Guinea (Conakry) (M.t.V.).

## Abstract

**Background and Objectives:**

Posttreatment radiologic deterioration of an irradiated high-grade (WHO grade 3–4) glioma (HGG) may be the result of true progressive disease or treatment-induced effects (TIE). Differentiation between these entities is of great importance but remains a diagnostic challenge. This study assesses the diagnostic value of conventional MRI characteristics to differentiate progressive disease from TIE in HGGs.

**Methods:**

In this single-center, retrospective, consecutive cohort study, we included adults with a HGG who were treated with (chemo-)radiotherapy and subsequently developed a new or increasing contrast-enhancing lesion on conventional follow-up MRI. TIE and progressive disease were defined radiologically as stable/decreased for ≥6 weeks or Response Assessment in Neuro-Oncology progression and histologically as TIE without viable tumor or progressive disease. Two neuroradiologists assessed 21 preselected MRI characteristics of the progressive lesions. The statistical analysis included logistic regression to develop a full multivariable model, a diagnostic model with model reduction, and a Cohen kappa interrater reliability (IRR) coefficient.

**Results:**

A total of 210 patients (median age 61 years, interquartile range 54–68, 189 male) with 284 lesions were included, of whom 141 (50%) had progressive disease. Median time to progressive disease was 2 (0.7–6.1) and to TIE 0.9 (0.7–3.5) months after radiotherapy. After multivariable modeling and model reduction, the following determinants prevailed: radiation dose (odds ratio [OR] 0.68, 95% CI 0.49–0.93), longer time to progression (TTP; OR 3.56, 95% CI 1.84–6.88), marginal enhancement (OR 2.04, 95% CI 1.09–3.83), soap bubble enhancement (OR 2.63, 95% CI 1.39–4.98), and isointense apparent diffusion coefficient (ADC) signal (OR 2.11, 95% CI 1.05–4.24). ORs >1 indicate higher odds of progressive disease. The Hosmer & Lemeshow test showed good calibration (*p* = 0.947) and the area under the receiver operating characteristic curve was 0.722 (95% CI 0.66–0.78). In the glioblastoma subgroup, TTP, marginal enhancement, and ADC signal were significant. IRR analysis between neuroradiologists revealed moderate to near perfect agreement for the predictive items but poor agreement for others.

**Discussion:**

Several characteristics from conventional MRI are significant predictors for the discrimination between progressive disease and TIE. However, IRR was variable. Conventional MRI characteristics from this study should be incorporated into a multimodal diagnostic model with advanced imaging techniques.

**Classification of Evidence:**

This study provides Class II evidence that in patients with irradiated HGGs, radiation dose, longer TTP, marginal enhancement, soap bubble enhancement, and isointense ADC signal distinguish progressive disease from TIE.

Diffuse gliomas are the most frequent adult-onset primary intra-axial malignancies^1^ and are classified in the WHO 2016 criteria according to histologic and molecular–genetic characteristics as grade 2, 3, or 4.^2^ High-grade gliomas (HGGs, WHO grade 3/4) include isocitrate dehydrogenase (IDH)–mutant astrocytomas, 1p/19q-codeleted oligodendrogliomas, and the more aggressive IDH–wild-type (WT) tumors. Despite aggressive treatment, survival is poor for most subtypes.

Post-treatment radiologic or clinical deterioration may be the result of true progressive disease or an effect of antineoplastic treatment. Such treatment-induced effects (TIE) are composed of tissue damage of the malignancy or the surrounding healthy cells, and include temporary pseudoprogression, typically developing within 3 to 6 months after radiotherapy, and late-delayed radionecrosis/radiation necrosis.^3-8^

On MRI, TIE and progressive disease can both reveal contrast enhancement (CE), mass effect, and edema. The accompanying clinical symptoms of progressive disease or TIE do not permit reliable discrimination.^4,9,10^ However, such a distinction is of great importance for further policy. Whereas progressive disease requires a change in antineoplastic treatment, TIE do not, although TIE may require specific symptom-directed therapy.

The current gold standard for distinction between progressive disease and TIE remains histopathologic examination of tissue obtained through invasive and costly brain biopsy. Furthermore, the interpretation of a histologic mixture of progressive disease and TIE can be difficult.

Noninvasive techniques for differentiation between progressive disease and TIE have been investigated. Multiple studies on advanced imaging, including perfusion MRI, have been conducted.^3,11^ However, perfusion imaging has a relatively low spatial resolution, is not always available for routine follow-up, and can be severely degraded by motion or susceptibility artefacts. The focus of this study lies therefore on conventional MRI characteristics, available from routine follow-up imaging. In retrospective cohort studies (18–169 patients), the combination of callosal involvement, multiple enhancing lesions^12^ and midline crossing,^12^ subependymal enhancement,^12-16^ appearance of a new enhancing lesion,^15^ and lower apparent diffusion coefficient (ADC) values^13,16,17^ were associated with progressive disease, whereas soap-bubble or Swiss cheese appearances were described in postradiation necrotic tumors.^18,19^ Due to this paucity of studies and the small sample sizes, a reliable differentiation between progressive disease and TIE by conventional MRI, the most applied and available method of follow-up, has not yet been investigated fully.

Our primary purpose was to assess the diagnostic value of conventional MRI characteristics to differentiate progressive disease from TIE in patients with a HGG and new or increased T1 CE after treatment. This study aims to provide a diagnostic model for determinants of TIE or progressive disease and to develop a prediction model to predict the disease status.

## Methods

### Standard Protocol Approvals, Registrations, and Patient Consents

The institutional Medical Ethical/Biobank Committee approved the use of patient data in the context of another study, for which patients had provided written informed consent for the use of MRI for response evaluation in future studies (protocol 16-342/16–229). For this retrospective analysis of a prospectively collected single-center cohort, all consecutive, adult patients diagnosed with a primary HGG by histopathology between January 1, 2011, and October 1, 2017, were eligible for inclusion. Inclusion criteria were (1) age ≥18 years, (2) treatment with radiotherapy of a HGG, (3) development of a new or increased CE lesion during routine follow-up with conventional MRI, which could be of any size, and (4) available reference test, consisting of histopathologic, radiologic, or clinical follow-up, which was used to diagnose progressive disease or TIE during multidisciplinary meetings.

### Image Acquisition

For the follow-up of glioma, we use a 1.5T or 3T MRI scanner (Philips Healthcare). Conventional MRI consisted of T1W MRI with and without contrast agent, T2-turbo spin echo/fluid-attenuated inversion recovery (FLAIR) MRI and ADC MRI, retrieved pre- and postradiotherapy and every 3 months thereafter. T2 FLAIR was acquired at a slice thickness of 4 mm with a 5 mm gap and an in-plane resolution of 0.41 mm. T1 after gadolinium was acquired in 3 directions at a slice thickness of 5 mm with a 6-mm gap and an in-plane resolution of 0.9 mm or in 3D at a slice thickness of 1.1 mm with a 1.1 mm gap. Patients were scanned with a 1.5 or 3T MRI based on availability. As we only used conventional MRI criteria, this was no confounding factor.

### Time Points and Definition of Outcomes

During follow-up, the first MRI showing a new or increased CE after ceasing radiotherapy was classified by viewing radiology reports and clinical records as the progression MRI (index test 2). The MRI prior to the progression MRI was considered as the baseline MRI (index test 1). TIE and progressive disease could be established radiologically (follow-up imaging), clinically, or histologically. Clinical TIE were defined as a stable or improved clinical status within a minimum period of 6 weeks. Radiologic TIE were defined as stable or decreased CE within a minimum period of 6 weeks with the latest scan showing stabilization being the reference scan. Histologic TIE were defined as TIE without viable tumor, with ≤1 mitosis, and progressive disease was defined as any viable, proliferating tumor at recurrence resection. Radiologic progressive disease was defined as further increase of the lesion resulting in progressive disease according to Response Assessment in Neuro-Oncology (RANO) criteria.^20^

### Clinical Determinants

The following baseline characteristics were collected: sex; age at first diagnosis; type of operation; histomolecular tumor type; type of treatment; received doses of radiation (equivalent dose in 2 Gy [EQD2]); time to progression (TTP), defined as the period between ceasing radiotherapy and progressive disease or TIE; and clinical deterioration at progression. Histopathology was interpreted according to the WHO 2007 grading system and was retrospectively updated to WHO 2016 criteria. For the WHO 2016 classification, the lesions were subdivided into (1) astrocytomas grade 3, IDH-mutant; (2) oligodendrogliomas grade 3, 1p19q codeleted and IDH-mutant; (3) astrocytomas grade 4, IDH-mutant; and (4) glioblastomas grade 4, IDH-WT.^2^ Gliomas with no available IDH status were excluded from this subgroup analysis. Gliomas grade 3 and 4 with a negative IDH immunostain (reflecting the absence of an IDH1 R132H mutation) were classified as a glioblastoma grade 4, IDH-WT, which includes a risk of misclassification of <10%.

Quantitative determinants were subdivided into groups: age (≤49, 50–59, 60–69, and ≥70 years), TTP (0–3, 3–5, and >5 months), tumor size at baseline (<1,000, 1,000–2,000, >2,000 mm^2^), and lesion growth (nonmeasurable disease at baseline, 0–10%, 10–25%, 25–50%, >50%). The categories for TTP were based on the timing of regular scanning (4 weeks postradiotherapy and then every ∼3 months). The timespan of 0–3 months represents the traditional interval for pseudoprogression,^10^ as incorporated in the original RANO criteria,^20^ whereas later publications also demonstrate the potential for pseudoprogression at later time points.^21^

The clinical information and index test results were accessible to the evaluator of the reference tests (junior researcher K.H.v.L.). In order to reduce observer bias, a blinded second observer (experienced neuro-oncologist T.J.S.), who was not informed about the index test results, checked all uncertainties and a random sample of the disease status. The follow-up period was fixed at a minimum of 6 weeks to have at least one additional follow-up MRI.

### Radiologic Determinants

We determined which conventional MRI characteristics should be assessed by researching the literature and group discussions. The observers discussed assessment of the MRI characteristics beforehand to optimize consistency. First, a junior researcher (K.H.v.L.) retrieved the MRI characteristics and then the outcome progressive disease or TIE and clinical characteristics from the electronic patient files. For the primary analyses (regression models), an experienced neuroradiologist (T.D.W.) assessed the MRI characteristics. A second experienced neuroradiologist (J.W.D.) re-assessed a sample of 100 patients for the interrater reliability (IRR) analysis. We estimated the sample size for the IRR with the sample size estimator for the Cohen Kappa statistic for a binary outcome N.cohen.kappa in R with the following values: probability that the raters will record a positive diagnosis = 0.5, true kappa statistic k1 = 0.7 based on previous literature,^15,22^ null hypothesis k0 = 0.4, 2-sided alpha = 0.05, and power = 0.8. We found n = 64, which we rounded up to 100 (package irr, R version 4.0.3). All 3 observers were blinded to the outcome at the time of MRI evaluation. We calculated an IRR coefficient (Cohen kappa^23^) between the junior researcher and the first neuroradiologist and between the 2 neuroradiologists. To reflect clinical practice, the neuroradiologists did not perform a consensus reading.

The following imaging characteristics were retrieved from the baseline MRI: tumor size and presence of nodular and callosal enhancement.

The following imaging characteristics were retrieved from the progression MRI: tumor size, enhancement crossing the midline,^12^ presence of necrosis, soap bubble enhancement,^18,19,24^ Swiss cheese enhancement,^18,19,24^ spreading wavefront pattern,^12,14,16,19^ T1 signal, FLAIR signal, and ADC signal, all 3 scored as hypo-, iso-, or hyperintensity compared with normal-appearing white matter. On the trace diffusion-weighted imaging b1000, the region of new or increased CE was scrutinized for the presence of visually apparent focal hyperintense areas. The ADC value in these focal areas was then compared with the ADC in normal white matter by region of interest measurement. On the T1 images, the region of new or increased CE was scrutinized for the presence of visually apparent focal hyperintensities compared with normal white matter.

Soap bubble enhancement was defined as small regions of necrosis.^24^ Swiss cheese enhancement was defined as more diffuse and larger regions of necrosis compared with soap bubble enhancement.^24^ Spreading wavefront pattern was defined as ill-defined borders of the enhancement instead of well-defined borders.^12^

**Figure 1:**
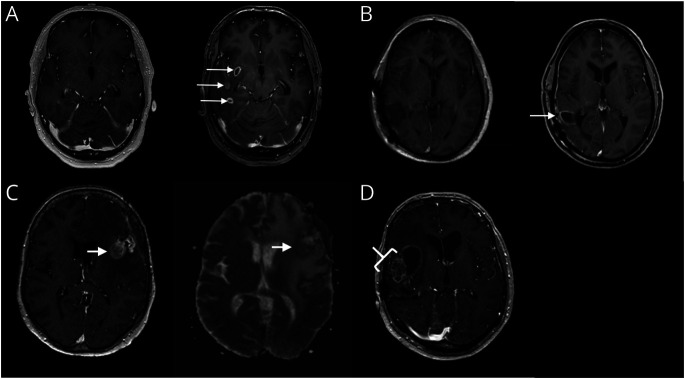
MRI Characteristics With Significant Predictive Value (A) A 64-year old man with an isocitrate dehydrogenase (IDH)–wild-type (WT) glioblastoma treated with radiotherapy. Left: Preoperative baseline MRI. Right: Follow-up MRI (93 days after baseline) with multiple new contrast enhancements. (B) A 58-year-old man with an IDH-WT glioblastoma treated with temozolomide-based chemoradiation. Left: Baseline MRI of the surgical cavity after resection. Right: Follow-up MRI (91 days after baseline) with increased marginal enhancement of the surgical cavity. (C) A 65-year old woman with a glioblastoma, IDH status unknown, treated with radiotherapy. Left: T1-MRI with contrast agent. Right: Isointense ADC signal compared with healthy white matter. (D) A 66-year old man with an IDH-mutated astrocytoma grade 4 treated with temozolomide-based chemoradiation: soap bubble enhancement (small regions of necrosis^17^).

The following characteristics were retrieved from comparing baseline and progression scan: percentage of tumor growth, new enhancement,^14,15^ multiple new enhancements,^12,16^ increased marginal enhancement surrounding the surgical cavity,^15^ new nodular enhancement,^14-16^ new callosal enhancement,^12,14-16^ new or increased enhancement in the septum pellucidum,^12^ new or increased subependymal enhancement,^12-16^ increased, stable, or decreased mass effect, and FLAIR abnormalities. Subependymal enhancement was defined as infiltration into the borders of the ventricles.^14^ Mass effect was scored in case of effacement of the sulci, compression of the ventricles, or midline shift. In case multiple new or increased contrast-enhancing lesions were detectable on the progression scan, these lesions were all reviewed separately. All measurements regarding tumor size were performed with RANO criteria^20^ and were noted separately for each lesion. Significant MRI characteristics are displayed in Figure 1 and nonsignificant MRI characteristics are assembled in eFigure 1 (links.lww.com/WNL/B943).

### Analysis

The analysis consisted of the following steps:

1. Missing value analysis
2. Univariable logistic regression analysis, generating odds ratios (ORs) with 95% CIs and *p* values, and a sensitivity, specificity, and positive and negative predictive value (PPV, NPV) for the items with the highest statistically significant OR
3. Checking for multicollinearity
4. Multivariable logistic regression analysis and development of a diagnostic model with ORs, 95% CIs, and *p* values; Hosmer and Lemeshow goodness of fit test was used for calibration of the model; diagnostic accuracy was computed by a receiver operating characteristic (ROC) curve and an area under the curve (AUC) with a 95% CI
5. Prediction model: model reduction was performed backwards with the SPSS bivariate logistic regression ENTER method manually and the likelihood ratio test with a threshold *p* value of 0.1 in order to develop a prediction model

We did not have any indeterminate index or reference tests. If a determinant was missing for >5% of cases, it was excluded from further analyses. In the case of <5% missing values, listwise deletion was performed. We chose not to perform multiple imputation because we had a very low number of missings, resulting in a limited advantage of imputation. Multicollinearity was investigated between determinants by computing a Pearson correlation coefficient. In case of a correlation coefficient >0.4 in absolute value, we established collinearity and selected the most relevant variable, based on available literature. ORs >1 indicate higher odds of progressive disease.

An outliers and extreme value analysis with histograms was conducted. Descriptive statistics of baseline characteristics consisted of frequencies or ranges with mean (SD) or median (interquartile range [IQR]). All statistical analyses were 2-tailed and performed in SPSS, version 25.0 and 26.0 (2017 and 2019, IBM SPSS Statistics). Significance levels were set at *p* < 0.05.

To evaluate the generalizability of the model, subgroup analysis was performed for (1) patients with an available histomolecular diagnosis according to the WHO 2016 criteria, (2) patients with a glioblastoma treated with temozolomide-based chemoradiation (representing the most prevalent subgroup of patients), (3) patients with a tumor size of more or less than 1,000 mm^2^ and more than 2,000 mm^2^, and (4) patients with a histologic reference test vs patients with a clinical/radiologic reference test (follow-up). The 2 different reference standards have their advantages and disadvantages, and histology is generally considered to provide the most definite proof of progressive disease or TIE, albeit in a selected subset and with risk of sampling error.^25^ For each subgroup, steps 1 to 4 were performed.

### Data Availability

Data not provided in the article because of space limitations may be shared (anonymized) at the request of any qualified investigator for purposes of replicating procedures and results.

## Results

### Patient Selection

**Figure 2:**
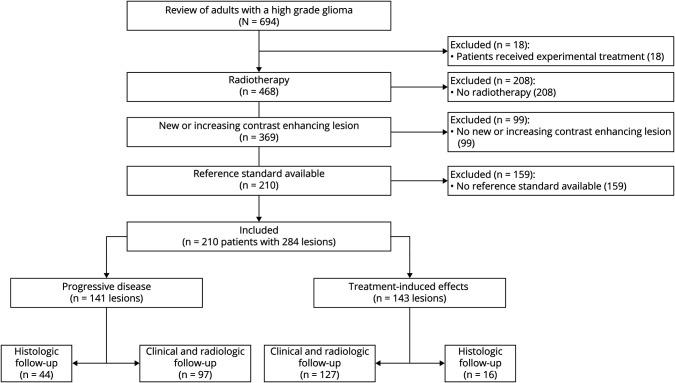
Patient Selection Process

Between January 1, 2011, and October 1, 2017, 694 patients received the diagnosis of a HGG. Of these patients, 210 with a total of 284 lesions were included in the present study. Figure 2 depicts the patient selection process. We documented the baseline characteristics in Table 1 per lesion as all statistical analyses were performed with the lesion characteristics. Among these lesions, 141 were finally classified as progressive disease and 143 as TIE. Of all lesions, 189 (67% of 284) belonged to a male patient. Ages ranged from 23 to 88 years. The reference test for 224 lesions was clinical or radiologic, whereas in 60 lesions histology was available. The subgroups for specific chemotherapeutics were too small to carry out statistical analysis and the group was dichotomized into a subgroup treated with radiotherapy only and a subgroup treated with radiotherapy and chemotherapy. The median follow-up period for patients with a radiologic reference test was 20 weeks (range 5.4–206.4) and for patients with a histologic reference test 7.9 weeks (range 1.4–71). In one patient, the radiologic follow-up was less than 6 weeks and we followed the patient clinically. This patient had his last follow-up scan showing progressive disease 5.4 weeks after the index test 2 and died 3 weeks later. No patient received bevacizumab at the moment of the index test 2.

**Table 1:**
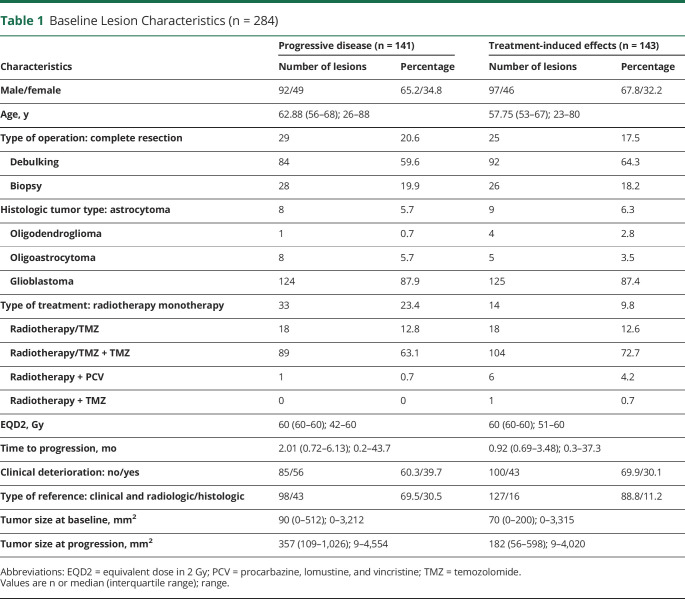
Baseline Lesion Characteristics (n = 284)

### Missing Values

The missing value analysis did not reveal any determinants with >5% missing values. A determinant with <5% missing values was a cerebral hemorrhage on the baseline scan of 1 patient with 5 lesions preventing the evaluation of the callosal enhancement criterion. In addition, 4 patients showed missing determinants, because their primary treatment had not been performed at our institution. MRI of the baseline scan and progression scan were available, but no diffusion or T1 scan without contrast agent. These missing values were considered unlikely to have influenced the outcome substantially.

### Diagnostic Model

In univariable logistic regression (significant results in Table 2), combination therapy compared with radiotherapy only (OR 0.36, 95% CI 0.18–0.70) and a higher radiotherapy dose predicted TIE (OR 0.67, 95% CI 0.48–0.93). Furthermore, a longer TTP (OR 2.29, 95% CI 1.29–4.07), necrosis (OR 2.02, 95% CI 1.06–3.85), and soap bubble enhancement (OR 2.21, 95% CI 1.30–3.76) correlated with progressive disease. Diagnostic test properties (95% CI) of necrosis included sensitivity 124 of 141 (88%) (81%–93%), specificity 31 of 143 (22%) (15%–29%), PPV 124 of 236 (53%) (50%–55%), and NPV 31 of 48 (65%) (51%–76%). Diagnostic test properties (95% CI) of soap bubble enhancement included sensitivity 112 of 141 (79%) (72%–86%), specificity 52 of 143 (36%) (29%–45%), PPV 112 of 203 (55%) (52%–59%), and NPV 52 of 81 (64%) (55%–73%).

**Table 2:**
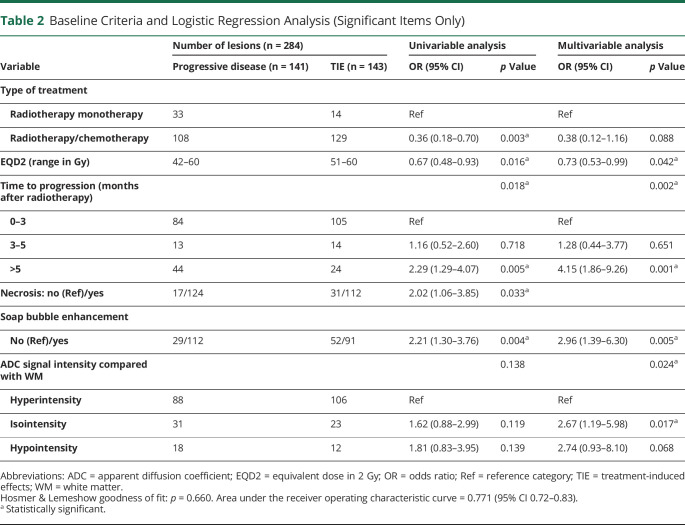
Baseline Criteria and Logistic Regression Analysis (Significant Items Only)

We found relevant multicollinearity between various variables influencing the eligibility for multivariable logistic regression analysis (eTable 1, links.lww.com/WNL/B943). As a result, the following determinants were rejected: tumor size at progression, enhancement crossing the midline, mass effect compared with baseline, necrosis, new enhancing lesion, and preexisting callosal enhancement. Based on previous literature and univariable analysis, 5 determinants were incorporated despite collinearity with another accepted determinant: type of treatment (collinear with radiation dose) and new callosal enhancement (collinear with new enhancement in the septum pellucidum and with new/increased subependymal enhancement).

Multivariable logistic regression analysis (Table 2) confirmed an association between a higher radiation dose and the risk of TIE (OR 0.73, 95% CI 0.53–0.99). In addition, a longer TTP (OR 4.15, 95% CI 1.86–9.26), soap bubble enhancement (OR 2.96, 95% CI 1.39–6.30), and isointense ADC signal compared with normal-appearing white matter (OR 2.67, 95% CI 1.19–5.98) showed an association with progressive disease. The calibration of the model was considered good with the Hosmer and Lemeshow goodness of fit test (*p* = 0.660) and the ROC curve showed an acceptable discrimination (AUC 0.771, 95% CI 0.72–0.83). Detailed results are shown in eTable 2 (links.lww.com/WNL/B943).

### Prediction Model

In reducing the full diagnostic model (as given in eTable 2, links.lww.com/WNL/B943) to generate the prediction model, 18 determinants were excluded during stepwise backward selection. The resulting prediction model (Table 3) contained 1 predictor of TIE (higher radiation dose) and 5 predictors of progressive disease (longer TTP, development of multiple new lesions, increased marginal enhancement, soap bubble enhancement, isointense ADC signal compared with normal-appearing white matter). The Hosmer and Lemeshow test showed a *p* value for the calibration of 0.947 and the AUC reached 0.722 (95% CI 0.66–0.78).

**Table 3:**
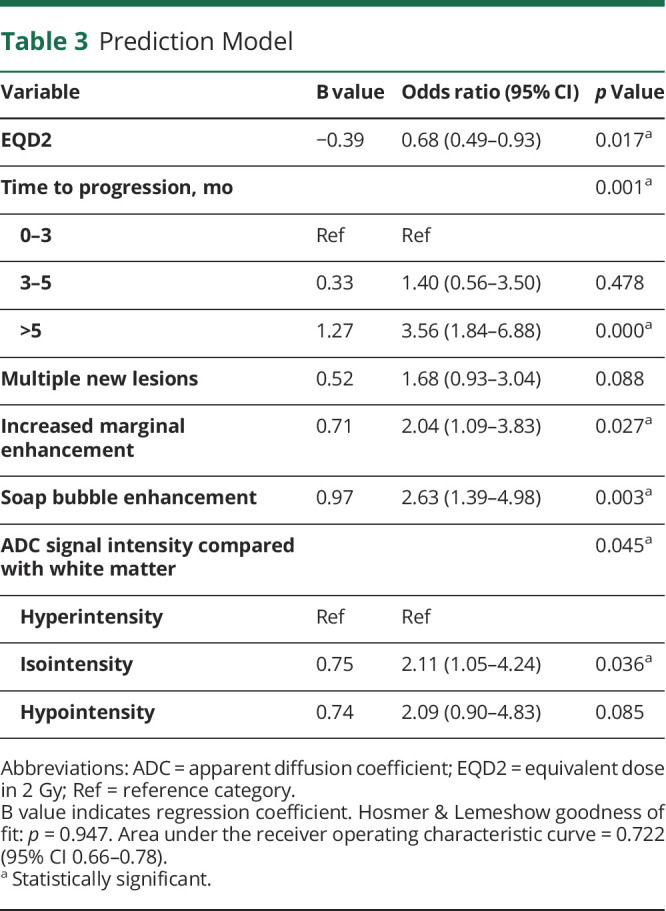
Prediction Model

### WHO 2016 Classification

A subset of 246 lesions could be (re-)classified according to WHO 2016 criteria (eTable 3, links.lww.com/WNL/B943). Univariable analysis for predictors of TIE vs progressive disease showed an association with age and 2 predictors of TIE (combination therapy compared with radiotherapy only and a higher radiotherapy dose) and 5 predictors of progressive disease (glioblastoma subtype, longer TTP, necrosis, soap bubble enhancement, and spreading wavefront enhancement). In multivariable analysis, the glioblastoma subtype (OR 16.03, 95% CI 2.48–103.43), a longer TTP (OR 6.78, 95% CI 2.50–18.34), and soap bubble enhancement (OR 3.05, 95% CI 1.23–7.58) predicted progressive disease and a higher radiotherapy dose (OR 0.14, 95% CI 0.03–0.57) was associated with TIE.

### Other Subgroup Analyses

Table 4 presents the significant items for the subgroup glioblastoma treated with temozolomide chemoradiation. eTable 4 (links.lww.com/WNL/B943) shows detailed results. Analysis showed an association with age and we found 2 additional predictors of progressive disease (longer TTP and marginal enhancement). In multivariable analysis, a longer TTP (OR 5.58, 95% CI 2.00–15.53), an increased marginal enhancement (OR 5.18, 95% CI 1.69–15.94), and an isointense ADC signal compared with white matter (OR 3.02, 95% CI 1.02–8.98) correlated with progressive disease.

**Table 4:**
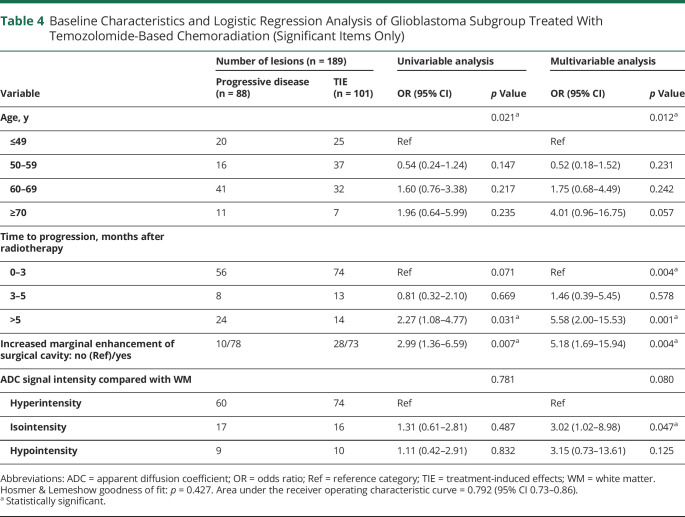
Baseline Characteristics and Logistic Regression Analysis of Glioblastoma Subgroup Treated With Temozolomide-Based Chemoradiation (Significant Items Only)

Regarding the subgroup tumor size below 1000 mm^2^ (eTables 5 and 6, links.lww.com/WNL/B943), univariable analysis revealed 2 predictors of TIE (radiotherapy/chemotherapy treatment and higher radiotherapy dose) and 6 predictors of progressive disease (TTP, a larger tumor size at baseline and at progression, necrosis, soap bubble enhancement, and spreading wavefront enhancement). In multivariable analysis, a higher radiation dose (OR 0.68, 95% CI 0.49–0.94) correlated with TIE. A longer TTP (OR 5.75, 95% CI 2.37–13.96) and soap bubble enhancement (OR 2.50, 95% CI 1.09–5.73) correlated with progressive disease. Due to small sample sizes, no reliable conclusion could be drawn for the subgroups with lesion sizes from 1,000 to 2000 mm^2^ (n = 18) or >2000 mm^2^ (n = 11).

Univariable analysis for the subgroup radiologic/clinical follow-up (n = 224) (eTable 7, links.lww.com/WNL/B943) revealed 2 predictors of TIE (type of treatment and EQD2) and 3 predictors of progressive disease (female sex, clinical deterioration, and a hypointense ADC signal compared with white matter). In multivariable analysis, EQD2 (OR 0.69, 95% CI 0.48–0.99) predicted TIE, and increased FLAIR abnormalities compared with white matter (OR 6.49, 95% CI 1.24–33.92), and a hypointense ADC signal compared with white matter (OR 4.31, 95% CI 1.19–15.55) correlated with progressive disease.

We performed a post hoc subgroup analysis of grade 3 gliomas and of the subgroup with a histologic reference test. Due to the low number of lesions (n = 35 for the nonglioblastomas and n = 60 for the histologic reference test), we performed an analysis with the significant items from the prediction model only (eTables 8 and 9, links.lww.com/WNL/B943). In the subgroup astrocytomas, oligodendrogliomas, and oligoastrocytomas, soap bubble enhancement correlated with progressive disease (OR 9.33, 95% CI 1.91–45.58). We performed no multivariable analysis. In the subgroup histologic reference test, soap bubble enhancement (OR 6.06, 95% CI 1.04–35.24) and marginal enhancement (OR 8.67, 95% CI 1.34–56.12) correlated with progressive disease in univariable and multivariable analysis.

### Interrater Reliability

IRR (Table 5) between the junior researcher and the neuroradiologist was slight to moderate. IRR between the 2 neuroradiologists revealed moderate to near-perfect agreement for the significantly predictive items of the primary analysis. IRR of other determinants was generally poor.

**Table 5:**
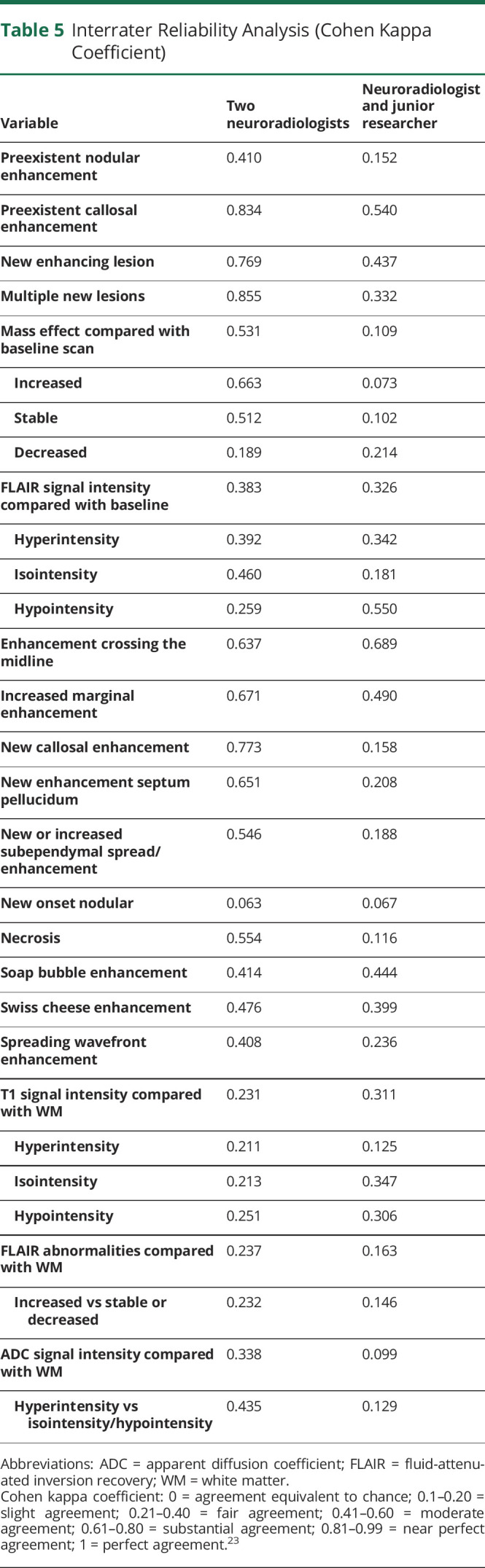
Interrater Reliability Analysis (Cohen Kappa Coefficient)

### Classification of Evidence

This study provides Class II evidence that in patients with irradiated HGGs, radiation dose, longer time to progression, marginal enhancement, soap bubble enhancement, and isointense ADC signal distinguish progressive disease from TIE.

## Discussion

In this single-center cohort study, the diagnostic value of conventional MRI characteristics was evaluated in their ability to differentiate progressive disease and TIE in patients with an HGG after radiotherapy. Various characteristics were independently associated with the disease status (progressive disease vs TIE). After incorporating these characteristics into a diagnostic and prognostic model, the calibration and accuracy of the model were considered good (AUC–ROC 0.722). Timing of occurrence of the progressive lesion was a strong predictor, with TTP beyond 5 months after radiotherapy having the highest predictive value (OR 3.56, 95% CI 1.84–6.88). This finding was replicated in all but 3 subgroups: over time, TIE becomes less and progressive disease becomes more likely, illustrating the inevitable tendency for progression in HGG.

Radiation dose, expressed as EQD2, was a predictor in all but 2 small subgroups. A higher radiotherapy dose has been linked earlier to more and purer TIE^26-29^; conversely, lower radiotherapy dose may be less effective, resulting in higher odds of progressive disease.

Soap bubble enhancement was described as a characteristic of TIE in previous literature.^18,19,24^ In contrast, in most of our analyses, soap bubble enhancement was a predictor for progressive disease. This could be the consequence of the vague definition of soap bubble, resulting also in a moderate IRR coefficient (0.414) despite an a priori specification. The target group could be of influence, because in the previously cited studies, a nonstandard, accelerated radiation scheme was used in one^18^ and brachytherapy in the other,^19^ which differed from our treatment schemes and could have increased the development of treatment-related necrosis. Furthermore, these authors included only patients with TIE and they did not study soap bubble enhancement in patients with progressive disease.^18,19^

A hypo- or isointense ADC signal compared with normal-appearing white matter was significantly associated with progressive disease in most multivariable analyses. This is in concordance with previous studies that reported lower ADC values in the recurrence group compared with the nonrecurrent group.^13,16,17^

After model reduction, multiple new lesions and increased marginal enhancement predicted progressive disease. The item multiple new lesions could reflect the poor prognosis of recurrent, multifocal diffuse gliomas. Multiple lesions and marginal enhancement were not independent, significant predictors in earlier, smaller studies.^12,14-16^

These results support the validity of the determinants EQD2, TTP, and soap bubble enhancement, which seem to be significant predictors. In 4 subgroups, small sample sizes may explain alternative findings. In contrast with previous literature, subependymal enhancement was not a predictor for progressive disease in our study.^12-16^ However, only 1 out of these 5 author groups noted (distant) subependymal enhancement as an independent predictor for early progression after multivariate analysis.^13^

Reliability between neuroradiologists was better than between a junior researcher and a neuroradiologist, but still variable, and poor for many characteristics. This suggests that these MRI items should be evaluated by a trained rater; alternatively, machine learning techniques could be used to overcome interrater variation. We found a Cohen Kappa for multiple new lesions (0.855) that was comparable to the Kappa for multifocal tumor recurrence from an earlier report (0.836).^22^ However, our kappa coefficients for other determinants were lower compared with previous literature.^15,22^ The imperfect IRR may contribute to the imperfect diagnostic value of some MRI characteristics.

Studies that tried to differentiate progressive disease and TIE with conventional MRI characteristics in large patient samples with treated HGGs are rare. The large sample size gathered over several years in our study constitutes a representative group of patients with HGG and baseline characteristics are comparable to those of a randomized trial on temozolomide-based chemoradiation.^30^ The robustness of findings was supported by the results of the subgroup analyses, which were largely in accordance with the results of our total study population.

Various limitations should be mentioned. First, the study design was single-center and retrospective. Treatment and follow-up were in line with (inter)national guidelines. However, at our institution, the normal scan interval starts within 4 weeks postradiation, followed by 3-monthly scans, rather than a longer interval. This could have led to a high degree of TIE, as these effects are more susceptible to appear during the first 3 months after radiotherapy and could otherwise have been missed.^9,10^

Second, patients with suspected radiologic progressive disease—wherein the treating physicians considered the diagnosis progressive disease to be obvious—were excluded from this study, which could have generated a selection bias. However, this choice was considered justified, because the purpose of this study was to identify predictive markers in the clinically relevant context of uncertainty between progressive disease and TIE.

Third, mixed lesions including parts with progressive disease as well as TIE were considered as progressive, since the progressive disease component guides prognosis and clinical management. Consequently, the radiologic characteristics analyzed in this study are probably more accurate in detecting progressive disease than TIE.

Lastly, we did not take dexamethasone use into account, because dexamethasone doses were inconsistently noted in electronic patient files. This could be responsible for an under- or overestimation of TIE, as dexamethasone is considered a symptomatic treatment of TIE, which may influence appearance and magnitude of MRI abnormalities.

The aim of our study was to find robust, consistent predictors of TIE or progressive disease, not to develop a generally applicable prediction model; such a model would seem incomplete without the use of perfusion MRI. Therefore, no external validation of this model will be performed. However, it remains challenging to discern progressive disease and TIE, even with perfusion MRI.^3,11,31,32^ Furthermore, perfusion MRI is not included in the RANO criteria or the standardized brain tumor imaging protocol for clinical trials and perfusion parameter cutoff values are not widely approved.^20,31,33^ Widely approved clinical parameters are even scarcer for the use of radiomics and associated machine learning/artificial intelligence techniques in the discrimination of progressive disease and TIE. Given these gaps of knowledge for perfusion MRI and radiomics, the optimal use of readily available features from clinical data and conventional MRI remains important.

We identified predictive characteristics from conventional MRI in distinguishing between true progressive disease and treatment-induced effects after radiotherapy of HGGs. IRR of some of these characteristics was poor. We suggest that these factors should be included in future prospective diagnostic studies on TIE, integrating conventional MRI items with contemporary techniques such as perfusion MRI and radiomics.

## Glossary

ADC: apparent diffusion coefficient
AUC: area under the curve
CE: contrast enhancement
EQD2: equivalent dose in 2 Gy
FLAIR: fluid-attenuated inversion recovery
HGG: high-grade glioma
IDH: isocitrate dehydrogenase
IQR: interquartile range
IRR: interrater reliability
NPV: negative predictive value
OR: odds ratio
PPV: positive predictive value
RANO: Response Assessment in Neuro-Oncology
ROC: receiver operating characteristic
TIE: treatment-induced effects
TTP: time to progression
WT: wild-type
